# Emerging Dissemination of *bla*_CTX‐M‐65_ in Bovine *E. coli* in Spain Associated With IncHI2 Plasmids

**DOI:** 10.1002/mbo3.70314

**Published:** 2026-05-20

**Authors:** Medelin Ocejo, Beatriz Oporto, Ana Hurtado

**Affiliations:** ^1^ Animal Health Department, NEIKER – Basque Institute for Agricultural Research and Development, Basque Research and Technology Alliance (BRTA) Bizkaia Science and Technology Park 812 L Derio Bizkaia Spain

**Keywords:** AmpC β‐lactamase producing *E. coli*, antimicrobial resistance (AMR), *bla*_CTX‐M‐65_, extended‐spectrum β‐lactamase (ESBL) producing *E. coli*, livestock, whole‐genome sequencing (WGS)

## Abstract

Food‐producing animals act as reservoirs of antimicrobial‐resistant bacteria, which may serve as sources of infection in humans. A selection of ESBL/AmpC‐producing *E. coli* isolates recovered from healthy (*n* = 37) and diseased animals (*n* = 26) were subjected to long‐read whole‐genome sequencing to characterize their genomic diversity, phylogeny, resistome, mobilome, and the genetic environment of antimicrobial resistance genes (ARGs). Isolates showed high genetic diversity (36 STs, seven phylogroups, 42 serotypes, 24 *fimH* types) and a diverse resistome, with CTX‐M enzymes as the most prevalent ESBL type and *bla*_CTX‐M‐14_ and *bla*_CTX‐M‐65_ predominating. Most ARGs were plasmid‐encoded (71.9%), with IncHI2 plasmids harboring the highest number of ARGs (8–20) and carrying complex multidrug resistance islands interspersed with integrons, transposons, and insertion sequences. All 15 IncHI2 plasmids were found exclusively in bovine isolates of multiple phylogroups (5) and STs (13), and most (13/15) carried *bla*_CTX‐M‐65_. This finding suggests a dissemination of this ESBL gene through a shared plasmid rather than a single dominant clone. This study reveals an emerging circulation of *bla*_CTX‐M‐65_ in bovine *E. coli* in Spain and identifies IncHI2 plasmids as major vehicles driving its spread. The presence of these plasmids in genetically diverse and potentially zoonotic linages reinforces their relevance from a One Health and food‐chain perspective.

## Graphical abstract

- The resistome in ESBL/AmpC‐producing *E. coli* in livestock was diverse and dominated by plasmid‐borne genes
- Detection of host‐independent dissemination of AMR determinants mediated by shared plasmid types
- First report in Spanish cattle of *bla*_CTX‐M‐65_ on IncHI2 plasmids, an under‐reported ESBL in Europe
- Identification of potentially zoonotic clones in livestock

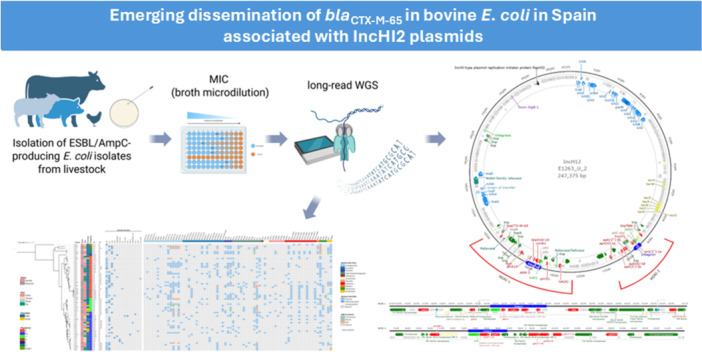

## 1 Introduction

Food‐producing animals are a relevant reservoir of resistant bacteria that can be disseminated to humans either by direct contact or indirectly via animal food‐products or the environment (Lazarus et al. 2015; McEwen and Collignon 2018). Resistance of Enterobacteriaceae to third‐ and fourth‐generation cephalosporins is a matter of concern because these agents are among the few remaining therapeutic options for severe infections caused by multidrug‐resistant (MDR) bacteria in humans (WHO 2019). In *Escherichia coli*, resistance to these antimicrobials (AM) is mainly mediated by the hydrolytic activity of extended‐spectrum β‐lactamases (ESBLs), plasmid‐mediated AmpC‐type β‐lactamases (pAmpC), and carbapenemases (CP), which are also relevant resistance mechanisms in other Enterobacterales. ESBL‐encoding genes mostly belong to the CTX‐M, SHV and TEM families (Castanheira et al. 2021), and pAmpCs are predominantly represented by the CMY family (Philippon et al. 2002). Additionally, a point mutation in the promoter can lead to increased expression of the *ampC* gene, resulting in higher levels of the AmpC β‐lactamase enzyme (Jacoby 2009).

Most ESBL and AmpC‐encoding genes are located on plasmids of different incompatibility (Inc) groups commonly associated with mobile genetic elements (MGEs) that can mobilize β‐lactam resistance determinants in addition to other co‐localized antimicrobial resistance genes (ARGs) that confer resistance to other classes of antibiotics (Partridge et al. 2018). Therefore, these elements play a key role in the acquisition and spread of antimicrobial resistance (AMR), favoring multidrug resistance through the co‐localization and mobilization of multiple resistance genes.

ESBL/AmpC‐producing *E. coli* are now widespread in humans as well as in food‐producing animals (Poirel et al. 2018; Dantas Palmeira and Ferreira 2020; Castanheira et al. 2021; Ewers et al. 2021) and may serve as important reservoirs of ARGs and as a potential source of resistance genes for other commensal or pathogenic Enterobacteriaceae. As such, their presence in food‐producing animals is considered a potential concern for public health, and they are therefore used as an indicator of AMR in surveillance programs. Surveillance of antimicrobial‐resistant pathogens is essential for understanding the local situation, and when combined with whole genome sequencing (WGS), it provides insights into the genetic basis of resistance mechanisms and supports early detection of emerging resistance.

In this study, the whole genome of a selection of ESBL/AmpC‐producing *E. coli* isolated from livestock (cattle, sheep, pigs, and chickens) in northern Spain was sequenced using Oxford Nanopore Technologies (ONT) to compare their genomic background and characterize their resistome. The main outcomes of such characterization are presented, highlighting the emergence of *bla*_CTX‐M‐65_ among bovine *E. coli* isolates mediated by IncHI2 plasmids.

## 2 Materials and Methods

### 2.1 Sample Collection

ESBL/AmpC‐producing *E. coli* isolates recovered from healthy (*n* = 37) and diseased animals (*n* = 26) were included in this study. Isolates from healthy animals were collected as part of an epidemiological surveillance study of AMR in food‐producing animals (cattle, sheep, pigs, and free‐range chickens) carried out at three slaughterhouses in northern Spain (Basque Country) between 2021 and 2023 (see Supporting Information S1: Note S1). Pure cultures of ESBL/AmpC‐producing *E. coli* from clinical cases were retrieved from our laboratory's strain repository, where they had been stored at −80°C. These clinical isolates were originally obtained from cattle in northern Spain, mostly between 2020 and 2023. They had been recovered from different types of samples (feces, milk, respiratory exudate, abomasum, small intestine or lung) from diseased animals that were submitted to the diagnostic service of the Department of Animal Health of NEIKER.

Prior to further characterization, all isolates included in the study were subjected to multiplex real‐time PCR for *E. coli* species identification (*uid*A gene) (Frahm and Obst 2003).

Isolates from slaughtered animals (one isolate from each positive pool: 12 cattle, 4 sheep, 13 pigs, 8 chickens) were selected to represent the diversity of animal sources and isolation rates, as well as the phenotypic resistance profiles as determined using the Sensititre® microdilution plate system (EUVSEC. 2 and EUVSEC. 3 panels, Thermo Fisher Scientific) (see Supporting Information S1: Note S1). All selected isolates originated from distinct farms, except for chicken isolates (8 isolates from 5 farms).

### 2.2 DNA Extraction and Whole Genome Sequencing (WGS)

For WGS, DNA from pure *E. coli* cultures was extracted using the NZYMicrobial gDNA Isolation Kit (NZYtech) according to the manufacturer's instructions. DNA purity and concentration were assessed with a Nanodrop ND1000 v2.5.3 spectrophotometer (ThermoFisher Scientific) and a Qubit 2.0 fluorometer (Invitrogen). For isolates from healthy animals, libraries were prepared with the Rapid barcoding kit (SQK‐RBK004) and sequenced on the flow cell FLO‐MIN106D (R9.4.1) from ONT. Bovine clinical isolates were sequenced using the Rapid barcoding kit 24 V14 (SQK‐RBK114.24) and FLO‐MIN114 (R10.4.1) flow cells. Sequencing was run on a MinION MK1C device (ONT) on Fast basecalling (Qscore > 8) to target ~650 Mb of passed reads per sample to ensure an approximate genome coverage of 100X.

### 2.3 Bioinformatic Analysis

Raw sequencing data were subjected to high‐accuracy basecalling (HAC) and filtered by quality using Guppy v.6.4.6 or Dorado v.7.1.4 (Qscore > 9). Adapters were then removed and filtered for length (> 1000 bp) using Porechop v.0.2.4 (Wick et al. 2017a) and Filtlong v.0.2.1 (https://github.com/rrwick/Filtlong), and fastq results were assembled *de novo* using the Unicycler v.0.5.0 tool (Wick et al. 2017b). Raw sequences and assembly descriptive statistics were assessed with Seqkit stats v.2.5.1 (Shen et al. 2016). Assembled genome completeness and contamination levels were estimated using CheckM v.1.2.2 (Parks et al. 2015).

The unassembled reads were used to search for resistance determinants associated with chromosomal single‐nucleotide point (SNP) mutations with the ResFinder v.4.5.0 tool (Bortolaia et al. 2020) and the PointFinder database v.4.0.1 (Zankari et al. 2017) (updated on 08‐03‐2023) at the Center of Genomic Epidemiology (CGE) platform (https://www.genomicepidemiology.org/). Multilocus Sequence Typing (MLST) profiling was also performed from unassembled reads using Krocus v.1.0.3 (Page and Keane 2018), allowing the assignment of a sequence type (ST) and a clonal complex (CC). The assembled genomes (contigs) were screened for ARGs (Resfinder and NCBI Database), disinfectant resistance genes (DRGs) (DisinfinderDB), virulence‐associated genes (VAGs) (ecoli_vf Database) and plasmid replicons (PlasmidFinder Database) using the ABRicate v.1.0.1 tool (T. Seemann, https://github.com/tseemann/abricate). Hits were filtered with a threshold of ≥ 90% coverage and ≥ 90% identity. Detected ARG and plasmid replicon variants were grouped and reported by major ARG families and plasmid incompatibility (Inc) groups, respectively. PlasFlow v.1.1.0 (Krawczyk et al. 2018) was used to predict the chromosomal and plasmid origin of contigs. The serogroup of strains was predicted with ABRicate using the Ecoh database to analyze the O antigens (*wzm, wzy, wzx* genes) and H antigens (*fli*C gene) of *E. coli* combined with the SerotypeFinder v.2.0.1 web tool (Joensen et al. 2015). Phylogroup assignment was performed *in silico* with the ClermonTyping online tool (Beghain et al. 2018), based on the Clermont PCR method. FimTyper v.1.0 (Roer et al. 2017) web tool was used to subtype the *fimH* gene, and species identity was confirmed using the Type Strain Genome Server (TYGS) (Meier‐Kolthoff and Göker 2019).

Pangenome analysis was conducted using Prokka (Seemann 2014) for genome annotation and Roary (Page et al. 2015) for gene clustering, both with default parameters. Core genome SNP‐based phylogeny was constructed using Parsnp v1.7.4 (Treangen et al. 2014), followed by maximum likelihood tree inference with RAxML v8.2.12 (Stamatakis 2014). Gene presence/absence matrices for AMR and virulence profiles were used to build hierarchical clustering dendrograms via hclust in R. Plasmid contigs were extracted with Seqtk, and plasmid replicon types, relaxase families, MPF types and predicted mobility were determined with MOB‐Typer v3.1.9 (Robertson and Nash 2018). IncH plasmids were functionally annotated using Bakta (v1.9.4; database v5.1.full) (Schwengers et al. 2021) and the resulting GFF3 annotation files were aligned with progressiveMAUVE (Darling et al. 2010) with Geneious to assess structural synteny and identify conserved backbone regions and variable modules. Plasmid double‐locus sequence typing (pDLST) (García‐Fernández and Carattoli 2010) was used to subtype IncHI2 plasmids based on the nucleotide sequences of two conserved loci, smr0018 and smr0199 (https://pubmlst.org/plasmid/). Complete insertion sequences (IS) were detected using ISEScan v.1.7.2.3 (Xie and Tang 2017). IntegronFinder v2.0.5 (Néron et al. 2022) was used to identify the number and locations of complete integrons and partial integrons containing either the insertion sites without the integrase nearby (CALIN) or containing the integrase without the insertion locations nearby (In0). Integron class was defined based on the integrase type determined by Bakta annotation or confirmed by blastn searches against the NCBI nucleotide database. Data visualizations were generated primarily using R (e.g., ggplot2, ComplexHeatmap) and genetic context was outlined with Geneious Prime 2025.1.1 (https://www.geneious.com).

### 2.4 Statistical Analysis

Descriptive statistics were used to summarize the distribution sequencing output, genomic characteristics, and the presence of genetic determinants of resistance (GDRs), VAGs, and DRGs. Continuous variables (e.g., genome completeness and contamination, total number of VAGs) were assessed for normality using the Shapiro–Wilk test. Non‐normally distributed data were analyzed using non‐parametric tests, such as the Mann–Whitney *U* test or Kruskal–Wallis test with post hoc pairwise comparisons using Dunn's test with FDR correction. All statistical analyses were performed using R (v4.3.0) with a significance threshold set at *p* < 0.05.

## 3 Results

### 3.1 ONT‐WGS Allowed High‐Quality Assemblies of Circularized Genomes and Plasmids, Revealing High Plasmid Content

The sequencing output varied by library kit and flow cell type. Isolates from healthy animals (SQK‐RBK004 + R9) produced a median of 74,167 reads (IQR = 68,080–80,152) with a median N50 of 15,293 bases (IQR = 13,086–15,923), while isolates from diseased cattle (SQK‐RBK114 + R10) yielded a higher read count (median = 162,518; IQR = 155,566–181,502) but lower N50 values (median = 6262 b; IQR = 5877–7479). Despite these differences, sequencing depth (median = 135X, IQR = 132–142) and total Mb sequenced per isolate (median = 677 Mb; IQR = 662–711) were comparable across groups (Supporting Information S2: Table S1).

Genome assemblies' completeness and contamination did not significantly differ between chemistries (*p* = 0.180 and *p* = 0.930, respectively) and showed high completeness (median 98.7%, IQR = 98.4%–99.1%) and low contamination (median 0.9%, IQR = 0.6%–1.4%). Circularized chromosomes were achieved for 82.5% (52/63) of isolates and the median genome size was 4,892,677 bases, with a GC content of 51%. A total of 227 plasmid contigs were assembled, of which 208 (91.6%) were circularized. Of them, 138 were detected in cattle isolates (100 diseased; 38 healthy), 44 in pigs, 31 in chickens, and 14 in sheep. The number of plasmids carried per isolate did not vary between host species (*χ* ^2^_kwallis_ = 0.28, *p* = 0.960) or cattle health status (W_Mann‐Whitney_ = 197.00, *p* = 0.200) and ranged from one to nine, with a median of three plasmids per isolate. The size of the plasmid contigs ranged from 0.7 to 289.7 kb, with a median of 90.7 kb (Supporting Information S2: Table S2).

### 3.2 Sequenced Isolates Represented a Genetically Diverse *E. coli* Population Across Host Species

**Figure 1:**
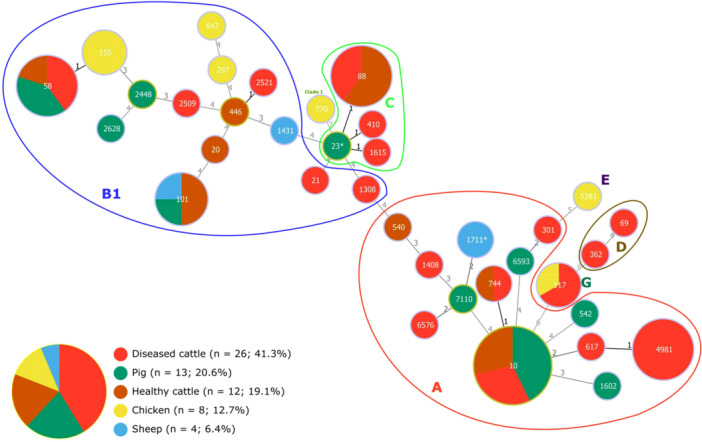
Minimum spanning tree (MST) based on MLST profiles (goeBURST, full MST) of ESBL/AmpC‐producing *E. coli* isolates from different livestock hosts. The MST shows the relationships between 63 *E. coli* isolates based on sequence type (ST), grouped by host source: cattle (diseased and healthy), pigs, chickens, and sheep. Each node represents a distinct ST, and the size of the node corresponds to the number of isolates. Colored sectors within nodes indicate the distribution of isolates from different hosts. The numbers on the connecting lines indicate the number of allelic differences between STs. Phylogroups are labeled and/or circled.

The 63 sequenced isolates were classified into seven phylogroups: A, B1, C, D, E, G, and clade I. Phylogroups A (39.7%, 25/63) and B1 (36.5%, 23/63) were the most prevalent, followed by C (12.7%, 8/63). Phylogroup B1 was the only one detected in all animal species, while phylogroup A was not identified in chickens (Figure 1). Chickens showed the highest phylogroup diversity, including uncommon phylogroups such as E, G, and clade I.

MLST analysis revealed 36 distinct STs assigned to nine clonal complexes (CCs). Most STs (75%, 27/36) were represented by a single isolate, with only four (ST10, ST58, ST88, ST4981) comprising more than five isolates (Figure 1). No ST was common to all four host species, but one type (ST101) was shared between cattle, sheep and pigs, and two (ST10 and ST58) were found in cattle and pigs. The diversity of ST profiles was the highest in chicken strains.

**Figure 2:**
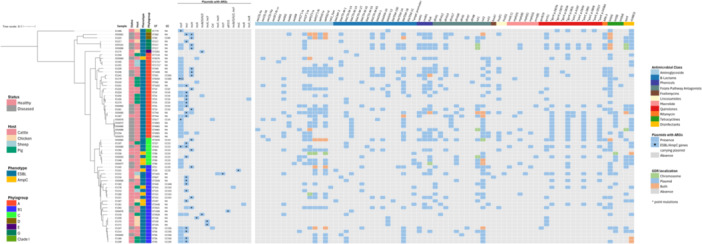
SNP‐based core genome phylogeny and heatmap of the distribution and localization of genetic determinants of resistance (GDRs) among ESBL/AmpC‐producing *E. coli* isolates from livestock. A maximum‐likelihood phylogenetic tree is shown on the left, annotated with sample ID, host, health status, MLST (ST, sequence type; CC, clonal complex), phenotype, and phylogroup. Plasmids carrying ARGs are also shown, with an asterisk denoting those encoding ESBL/AmpC genes. The heatmap displays the presence and genomic location of GDRs grouped by antimicrobial classes (indicated by color‐coded bars above the gene names and the legend). GDR localization is represented by color as per legend. Asterisk in the name of a GDR indicate chromosomal point mutations.

Forty‐two distinct O:H serotypes were identified, including serotypes with recognized zoonotic potential such as O26:H11 (E1242, ST21) and O45:H2 (E1241, ST301). Fifty‐six isolates belonged to 24 *fimH* subtypes (seven isolates were non‐typable), with *fimH54* and *fimH32* predominating (Figure 2).

A total of 491 VAGs were identified, and 92.9% were chromosomally encoded (Supporting Information S2: Figure S1). Each isolate contained between 123 and 276 VAGs. Total VAG content did not significantly differ by host species (*χ²* = 4.77, *p* = 0.190) or by cattle health status (*W* = 170.5, *p* = 0.660), but did vary by phylogroup (*χ²* = 24.94, *p* < 0.001), with phylogroups B1, C, and D showing higher counts than phylogroup A (median: A = 169, B1 = 195, C = 233.5, D = 243). Five *E. coli* isolates from cattle carried the Shiga‐toxin genes *stx1* and *stx2* or the intimin *eaeA* gene (Supporting Information S2: Figure S1).

Core‐genome SNP phylogeny was broadly consistent with phylogroup and ST assignments, but neither host health status nor resistance profile correlated with the phylogenetic clustering (Figure 2).

### 3.3 *bla*_CTX‐M‐14_ and *bla*_CTX‐M‐65_ Were the Predominant ESBL‐Associated Genes in a Diverse *E. coli* Resistome

Resistome analysis identified 70 unique GDRs, including 59 acquired ARGs and 11 SNPs, associated with resistance to 10 AM classes, as well as two genes encoding resistance to disinfectants (Figure 2).

The most frequently acquired resistance determinants identified were associated with β‐lactams, aminoglycosides, folate pathway antagonists, tetracyclines, and phenicols. Resistance to β‐lactams was mainly encoded by *bla*_TEM_ and *bla*_CTX‐M_ gene variants. CTX‐M enzymes were the most prevalent ESBL type, with CTX‐M‐14 and CTX‐M‐65 predominating among the seven CTX‐M variants detected. In this dataset, *bla*_CTX‐M‐14_ occurred mainly in pig isolates, whereas *bla*_CTX‐M‐65_ was detected only in bovine isolates, with both genes being consistently carried by plasmids. Two bovine isolates carried two *bla*_CTX‐M_ gene variants on separate plasmids. In chicken isolates, *bla*_SHV‐12_ was the most frequent ESBL determinant. AmpC‐associated resistance was mainly encoded by a point mutation in the chromosomal *ampC* promoter (*n* = 7) and, less frequently, by the *bla*_CMY‐2_ gene (*n* = 2). Beyond β‐lactam resistance, plasmid‐ and chromosome‐associated GDRs explained the high levels of phenotypic resistance to trimethoprim/sulfamethoxazole, tetracycline, aminoglycosides, and phenicols, while quinolone resistance was largely associated with chromosomal mutations in *gyr*A, *par*C, and *par*E, sometimes together with plasmid‐mediated *qnr* genes (Figure 2). GDRs were more widespread in isolates from cattle (Supporting Information S1: Note S2) and in diseased cattle compared to healthy cattle (Supporting Information S1: Note S3).

Genes associated with resistance to disinfectants were detected in 33 of the 63 genomes (Figure 2). The *sitABCD* operon, associated with resistance to hydrogen peroxide, was detected in 16 isolates (25.4%), *qac*Δ*E*, linked to resistance against quaternary ammonium compounds, was found in 4 isolates (6.3%), and co‐occurrence of both genes was observed in 13 isolates (20.6%). The *sitABCD* operon was mainly plasmid‐borne (always IncF plasmids), with no clear association with nearby ARGs. In contrast, *qac*Δ*E* was predominantly chromosomally encoded and consistently integrated within a class I integron gene cassette together with additional ARGs, and co‐localized with metal resistance genes (MRG) operons (*merACDEPRT*, *stiP*), forming multi‐resistance clusters.

### 3.4 The Resistome of ESBL/AmpC‐Producing *E. coli* Was Dominated by Plasmid‐Borne Genes, With IncF and IncH Plasmids Carrying the Highest Number of ARGs

**Figure 3:**
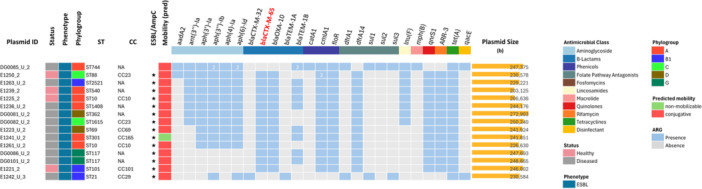
Distribution of antimicrobial resistance genes (ARGs) in IncHI2 plasmids from ESBL/AmpC‐producing *E. coli* isolates. Isolate metadata (host species, health status, sequence type (ST), phylogroup) and plasmid metadata (incompatibility group, predicted mobility and plasmid size) are shown. The heatmap shows the presence of ARGs, grouped by antimicrobial class (color‐coded bar above and legend). Orange bars on the right represent plasmid size (bp).

A total of 71.9% of ARGs were located on plasmids, including most of the ESBL‐encoding genes (46/56, 82.1%) (Figure 2). Overall, 15 different plasmid replicon types were detected, including a few multi‐replicon plasmids. The most frequently detected incompatibility (Inc) groups were IncF (*n* = 51), IncI (*n* = 30), Col (*n* = 16) and IncH (*n* = 15) (Table 1). Plasmids carrying ARGs (93 of the 227) were detected in all isolates except three (95.2%), each isolate carrying between one and three different ARG‐harboring plasmids of variable sizes (3–290 kb) (Supporting Information S2: Table S2). Most of them (90.3%) were predicted to be conjugative, 4.3% mobilizable, and 5.4% non‐mobilizable. The heatmap (Supporting Information S2: Figure S2) illustrates the distribution of ARGs across plasmids and shows high heterogeneity among replicon types and hosts. For example, IncI plasmids harbored the highest diversity of ESBLs, carrying *bla*_CTX‐M‐14_, *bla*_SHV‐12_, *bla*_CTX‐M‐32_, and *bla*_CTX‐M‐1_ genes (total *n* = 21). In contrast, IncHI2 plasmids predominantly carried bla_CTX‐M‐65_ (*n* = 13), occasionally accompanied by *bla*_CTX‐M‐32_ (n = 1) (Figure 3 and Supporting Information S2: Figure S2). Other plasmid types, including IncF, IncB/O/K/Z, IncX, IncY, and p0111, occurred sporadically and carried various *bla*_CTX‐M_ variants or *bla*_SHV‐12_. Hierarchical clustering of plasmids based on shared ARG profiles revealed notable diversity in ARG content (Supporting Information S2: Figure S2), with IncHI2 and IncF harboring the highest number of ARGs per plasmid (up to 20 and 15, respectively), almost covering all detected AM classes. In contrast, Col‐type plasmids (the smallest in size) and those lacking a known replicon, generally carried few or no ARGs.

**Table 1:** Summary of plasmid replicon types identified among *Escherichia coli* isolates from livestock. Plasmids were categorized according to incompatibility (Inc) type and characterized by their mean size (bp), total number detected (N), proportion carrying at least one antimicrobial resistance gene (ARG), and the range of ARGs per plasmid.

Plasmid type	Size (mean, bp)	*N*	Plasmids w/ARG *n* (%)	nARGs (range)
IncF	121,773	51	35 (68.6%)	0–13
IncI	101,140	30	25 (83.3%)	0–6
Col	5,134	16	2 (12.5%)	0–2
IncH	237,840	15	15 (100.0%)	8–20
IncX	35,172	12	4 (33.3%)	0–4
IncB/O/K/Z	107,618	7	3 (42.9%)	0–4
IncY	96,637	6	1 (16.7%)	0–8
p0111	103,107	4	1 (25.0%)	0–1
IncX, IncY	145,738	2	2 (100.0%)	2
IncB/O/K/Z, IncF	289,691	1	1 (100.0%)	2
IncC	95,159	1	1 (100.0%)	6
IncF, IncH	215,611	1	1 (100.0%)	10
IncF, IncR	37,635	1	1 (100.0%)	2
IncR	46,490	1	1 (100.0%)	5
IncP	49,422	1	0 (0.0%)	0
Unknown replicon	36,284	78	0 (0.0%)	0
**Total**	**83,337**	**227**	**93 (41.0%)**	**0–20**

### 3.5 IncHI2 Plasmids Drive the Emergence of *bla*_CTX‐M‐65_ in Bovine *E. coli* in Spain

IncH plasmids (subgroup IncHI2) were all hosted by isolates from cattle (11 diseased and 4 healthy) distributed among the 5 phylogroups identified in cattle (A, B1, C, D, G) and assigned to 13 different STs. IncHI2 plasmids were the largest in size (median size *ca.* 250 kb) and carried the higher number of ARGs (8–20 ARGs, that conferred resistance to 6–9 AM classes) (Figure 3).

A comparative genomic analysis using MAUVE revealed a high structural similarity, with 10 major collinear blocks shared by most of the 15 IncHI2 plasmids (Supporting Information S2: Figure S3). Still, plasmid double‐locus sequence typing (pDLST) assigned them to either pST‐4 (*n* = 2), pST‐3 (*n* = 10), or untyped but with pST‐3 as the nearest match (n = 3). pST‐3 and related plasmids (*n* = 13) carried *bla*_CTX‐M‐65_, while pST‐4 plasmids carried *bla*_CTX‐M‐32_ (E1242_U_3) or did not carry any ESBL‐encoding gene (DG0085_U_2). A core set of ARGs was recurrently detected in the IncHI2 plasmids, which included *tet*(A) (15/15), *floR* and *cmlA1* (14/15), *bla*_CTX‐M‐65_, *bla*_OXA‐10_, ARR‐3, *qnrS1*, and *dfrA14* (13/15 each), conferring resistance to tetracyclines, phenicols, β‐lactams, rifamycin, fluoroquinolones, and trimethoprim. Aminoglycoside resistance was encoded by *aph(6)‐Id* and *aph(3″)‐Ib* (12/15), *aph(4)‐Ia* and *aph(3′)‐Ia* (11/15), and *ant(3″)‐Ia* (8/15). Other genes such as *aadA2*, *catA1*, *mph*(B), *sul1/2/3*, and *bla*_CTX‐M‐32_ were sporadically detected.

All IncHI2 plasmids harbored at least one MDR island (MDRI), interspersed with MGEs like integrons, transposons, and ISs. In total, 20 complete class 1 integrons and five CALINs were identified. Complete integrons were present in 14 plasmids (all except DG0081_U_2), with up to three distinct integron structures per plasmid. Plasmids were densely populated with ISs, totaling 218 elements from nine IS families, the most abundant being the IS6 family (Supporting Information S2: Table S3). ARGs were often embedded within composite transposons flanked by IS elements.

**Figure 4:**
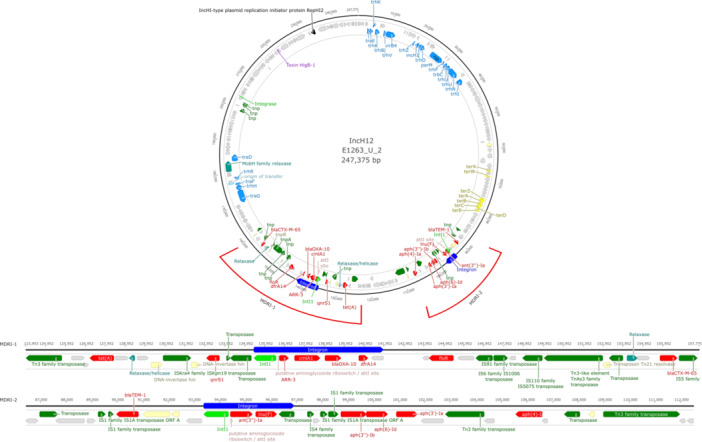
Genetic map of the representative IncHI2 plasmid E1263_U_3, illustrating the conserved arrangement of the two predominant multidrug resistance islands (MDRIs). The circular map depicts annotated coding sequences (CDSs) colored by functional category: conjugative elements (blue), antimicrobial resistance genes (red), insertion sequences or transposases (green), toxin‐antitoxin systems (purple), heavy metal resistance genes (yellow) and other CDSs (gray). Red lines indicate the locations of two major MDRIs (MDRI‐1 and MDRI‐2), expanded below for detailed linear visualization.

Two main MDRI were identified (Figure 4). MDRI‐1, present in all pST‐3 and related plasmids (*n* = 13), included a conserved class 1 integron carrying the ARG cassette *ARR‐3, cmlA1, bla*_*OXA‐10*_ and *dfrA14*. This integron was consistently flanked upstream by *qnrS1* and *tet*(A), and downstream by *floR* separated by more than 10 kb from *bla*_CTX‐M‐65_ gene. MDRI‐2, present in 8 pST‐3 and one pST‐4 plasmids, was characterized by a class 1 integron carrying the ARG cassette *ant(3″)‐Ia* and *lnu*(F), typically preceded by *bla*_TEM‐1_ and followed by a compact aminoglycoside resistance cluster (*aph(3″)‐Ib, aph(6)‐Id, aph(3′)‐Ia*, and *aph(4)‐Ia*). The two pST‐4 IncHI2 plasmids (DG0085_U_2 and E1242_U_3) carried another MDRI where the *qac*Δ*E* gene was present as part of a class I integron and co‐localized with MRG operons (*merACDEPRT*, *stiP*). All IncHI2 plasmids encoded tellurite resistance operons (*terZABCDE* and *terWX*), although these loci were positioned outside of MDR regions.

All IncHI2 plasmids encoded a complete Type IV Secretion System (T4SS), facilitating conjugative transfer. Key conjugation‐related genes such as *tra*, *trh*, *virB4*, and associated modules (*TraE/F/N/G*, *TrbC*, *ParM*) were conserved across the plasmid set.

## 4 Discussion

This study aimed to characterize the genomic diversity and resistome of extended‐spectrum cephalosporin‐resistant *E. coli* in livestock in northern Spain. The use of ONT sequencing enabled the assembly of complete, circularized chromosomes and plasmids, offering high‐resolution insights into their phylogenetic relationships and the distribution of GDRs, DRGs and VAGs, as well as their genetic environment and mobilome. The selection of isolates sequenced represented a high genetic diversity, with 36 distinct STs distributed across seven different phylogroups, 42 different serotypes, and 24 *fimH* types. It included InPEC/Commensal phylogroups (A, B1, C) represented by isolates recovered from all the animal sources analyzed and ExPEC (D, E, G, clade I) isolates from chicken and cattle. Pangenome analysis derived phylogeny was consistent with phylogroup and ST assignments. Genomic analysis further confirmed differences in GDR and VAG content.

As described elsewhere (Seiffert et al. 2013; Tello et al. 2020; EFSA/ECDC 2025), ESBL‐encoding genes were more frequently detected than AmpC‐encoding genes in the *E. coli* strains analyzed. A mutation in the promoter region of the chromosomal *ampC* gene predominated over the carriage of plasmid‐mediated AmpC‐encoding genes in all hosts except chicken, which agrees with previously reported data (EFSA/ECDC 2025). Genes encoding CTX‐M enzymes were the most prevalent type of ESBL, but the variants that predominated differed from those reported in Europe (EFSA/ECDC 2025). In this study, *bla*_CTX‐M‐14_ and *bla*_CTX‐M‐65_ (both of CTX‐M‐9 group) predominated, while CTX‐M‐1 group (particularly *bla*_CTX‐M‐15_) is spread in many other countries. In Spain as well as in China, South‐East Asia, South Korea, and Japan, group 9 variants, and especially *bla*_CTX‐M‐14_, predominate (Bevan et al. 2017). The high prevalence of *bla*_CTX‐M‐65_ observed here in *E. coli* isolated from cattle was unexpected, as this variant had not been previously detected in other studies carried out in the region (Tello et al. 2020, 2022) and remains relatively uncommon worldwide (Yu et al. 2024). Thus, only 0.6% (3,007/513,959) of *E. coli* genomes in the NCBI Pathogen Detection Database (accessed 25/11/25) carried the *bla*_CTX‐M‐65_ gene, of which just 65 of them originated from cattle, three of them from Spain. However, it has recently become one of the dominant CTX‐M types in animal isolates in China (Wang et al. 2018) where it is the most common ESBL type in human infections (Zhou et al. 2015). The emergence of CTX‐M‐65‐producing *E. coli* has also been recently reported in beef and pork meat in Portugal (Leão et al. 2021).

The majority of ARGs detected in this study were plasmid located, including all *bla*_CTX‐M‐14_, *bla*_CTX‐M‐65_, *bla*_SHV‐12_, and *bla*_TEM‐1A_ genes. Our findings indicated that IncI and IncHI2 plasmids were the main carriers of ESBL determinants, particularly *bla*_CTX‐M‐14_ and *bla*_CTX‐M‐65_, respectively. Among the different plasmid replicon types identified, IncHI2 plasmids were of particular concern as they carried the broadest repertoire of ARGs, including *bla*_CTX‐M‐65_, which were consistently present in IncHI2 plasmids. While IncHI2 plasmids are known vectors of resistance, their combination with *bla*_CTX‐M‐65_ remains rare and occurs more frequently in *Salmonella* than in *E. coli* (Brennan et al. 2016; Wang et al. 2023; Algarni et al. 2024; Yu et al. 2024). At the time of manuscript preparation (25/11/25), only 76 IncHI2 plasmids carrying *bla*_CTX‐M‐65_ were registered in the PLSDB database (Molano et al. 2025), with *E. coli* representing 40.8% of them (second to *Salmonella enterica*, which represented 46.1%) and only two records associated with cattle. Previous studies have identified plasmids of the IncF family as the main carriers of *bla*_CTX‐M‐65_ in *E. coli* (Yu et al. 2024). In this study, several lineages of *E. coli* acquired similar antimicrobial resistance traits through these IncHI2 plasmids, including three STEC isolates and one isolate belonging to the ST69 pandemic ExPEC lineage (phylogroup D; fimH27; serotype O15:H18). The detection of this plasmid type across multiple *E. coli* phylogroups, serovars, and STs indicates that the spread of *bla*_CTX‐M‐65_ is not restricted to a single lineage but is a more widespread phenomenon. This suggests that the spread of the CTX‐M‐65 enzyme is associated with the proliferation from various sources or clones, rather than a single dominant clone as described for CTX‐M‐9 and CTX‐M‐14 (Cantón and Coque 2006).

The IncHI2 plasmids characterized in this study harbored different class 1 integrons carrying multiple ARG cassettes flanked by a dense array of ISs, which together facilitate the capture, stabilization, and rearrangement of resistance determinants and enhance their dissemination and long‐term persistence (Cain and Hall 2012; Fang et al. 2018; Gillings et al. 2008). The two MDRI configurations most frequently observed (MDRI‐1 and MDRI‐2), which often coexisted on a single plasmid, highlight the modular nature of resistance acquisition. MDRI‐1 contained a cassette of ARGs that altogether conferred resistance to five classes of clinically relevant AMs in an arrangement that resembles In1459 integrons as reported in both clinical and environmental *Enterobacteriaceae* (Paskova et al. 2018). MDRI‐2 carried a different class I integron, harboring an aminoglycoside and lincosamide gene flanked by *bla*_TEM‐1_ and a cluster of five other aminoglycoside resistance genes. The *bla*_TEM‐1_ gene is frequently found adjacent to class 1 integrons (Wang et al. 2023), and similar tandem arrangements of aminoglycoside‐modifying enzyme genes have been described in *E. coli*, significantly broadening resistance to multiple aminoglycosides (Lindemann et al. 2012; Wellner et al. 2024). The conjugative nature of IncH plasmids makes them particularly concerning because they can act as vectors for transmission of the ARGs, DRGs, and MRGs they carry, leading to the spread of a wide range of resistance elements in a single transfer event (Fang et al. 2018; Dionisio et al. 2019). The high recombination potential, the capacity to integrate multiple resistance mechanisms, and their broad host range (Algarni et al. 2024), make IncHI2 plasmids critical vehicles for AMR dissemination.

One example of how MGEs contribute not only to the dissemination but also to the chromosomal stabilization of resistance genes is the consistent detection of *qac*Δ*E* within class 1 integrons, found on plasmids but mostly integrated into the chromosome. The proximity of ISs upstream or downstream of these integrons suggests past mobilization events, now stabilized in the chromosome (Partridge et al. 2001; Tamamura et al. 2013). The consistent co‐localization of *qac*Δ*E* with *sul1* and aminoglycoside or trimethoprim resistance genes within class 1 integrons supports previous findings (Hu et al. 2016; Li et al. 2020), and suggests that prolonged and improper use of quaternary ammonium compounds may co‐select for broader AMR even in the absence of direct antibiotic pressure and promote the maintenance of resistance clusters (Buffet‐Bataillon et al. 2012; Wales and Davies 2015; Davies and Wales 2019). Importantly, in several isolates, *qac*Δ*E* was also co‐located with *mer* operon genes conferring resistance to mercury, further supporting the role of class 1 integrons and associated MGEs in the co‐selection of disinfectant, antibiotic, and metal resistance (Hazen et al. 2017; Enany et al. 2019). The other DRG detected, the *sitABCD* operon, exhibited a distinct genomic distribution. This operon, which contributes to iron uptake and resistance to oxidative stress, was commonly located on IncF plasmids but unlinked to neighboring ARGs, as reported elsewhere (Díez de los Ríos et al. 2025). This is consistent with previous work indicating that *sitABCD* may be maintained independently of resistance co‐selection due to its role in enhancing bacterial fitness in iron‐limited environments (Phan et al. 2015).

## 5 Conclusion

This study provides a high‐resolution genomic overview of ESBL/AmpC‐producing *E. coli* circulating among livestock in northern Spain, including isolates associated with both clinical cases and healthy animals. The co‐existence of extensive MDRIs on plasmids as well as chromosomally integrated ARGs, DRGs and MRGs reveals a complex and multifactorial resistance mechanism in these isolates. The results showed a widespread distribution in bovine isolates of *E. coli* belonging to multiple phylogroups and STs of the *bla*_CTX‐M‐65_ gene, a variant rarely reported in cattle both nationally and globally, which appears to be spreading through IncHI2 plasmids that harbor complex MDRIs rich in ARGs, integrons, and ISs. The presence of *bla*_CTX‐M‐65_ in clones with known zoonotic potential (e.g. ST10, ST69, ST88, ST101, and ST117), combined with the capacity of IncHI2 plasmids to transfer across bacterial species and hosts, adds further relevance to public health, especially considering the use of antimicrobials in livestock and the risk of spillover through the food chain.

## Author Contributions

**Medelin Ocejo:** data curation, formal analysis, methodology, validation, writing – original draft, visualization, writing – review and editing. **Beatriz Oporto:** data curation, formal analysis, methodology, writing – review and editing, investigation, validation. **Ana Hurtado:** data curation, conceptualization, formal analysis, validation, funding acquisition, writing – original draft, project administration, supervision, writing – review and editing.

## Ethics Statement

In accordance with the local legislation and institutional requirements, ethical approval was not required for samples collected at the slaughterhouse during routine carcass processing. Written informed consent was not obtained from the owners for the participation of their animals in this study because they are aware that all animals may be subject to random sampling at the slaughterhouse for disease surveillance, as implemented under the coordination of the public health department. Samples from diseased animals were collected by veterinary practitioners strictly following Spanish ethical guidelines and animal welfare regulations (Real Decreto 53/2013). The collection of this material, being considered a routine veterinary practice, did not require the approval of the Ethics Committee for Animal Experimentation. At the time of sample submission, farm owners gave their consent for the use of samples for research purposes, in accordance with the general terms and conditions for the provision of specialized analytical services. The researchers were never in contact with the animals from which the samples were collected.

## Conflicts of Interest

The funding source had no involvement in the study design, the collection, analysis and interpretation of data, the writing of the report, or in the decision to submit the article for publication. The authors have no competing interests.

## Supporting information

Supporting File 1

Supporting File 2

## Data Availability

The data that support the findings of this study are openly available in European Nucleotide Archive (ENA) at https://www.ebi.ac.uk/ena/browser/view/PRJEB104939. All raw metagenomic sequencing data generated in this study have been deposited in the European Nucleotide Archive (ENA) under the Study Accession number PRJEB104939. Reads Accession numbers range from ERR16001388 to ERR16001450 (Supporting Information S2: Table S2).
