# Supplementary material for: Emerging Dissemination of bla CTX‐M‐65 in Bovine E. coli in Spain Associated With IncHI2 Plasmids
Source: Microbiologyopen. 2026 May 20;15(3):e70314. doi: 10.1002/mbo3.70314 (PMC13239750; doi:10.1002/mbo3.70314)
Supplement: Supplementary file 1 — Supporting File 1 [file MBO3-15-e70314-s001.pdf]

Supplementary Notes S1 – S3

Emerging dissemination of *bla*$_{CTX-M-65}$ in bovine *E. coli* in Spain associated with IncHI2 plasmids

Medelin Ocejo, Beatriz Oporto, Ana Hurtado[#]

Animal Health Department, NEIKER – Basque Institute for Agricultural Research and Development, Basque Research and Technology Alliance (BRTA), Bizkaia Science and Technology Park 812L, 48160 Derio, Bizkaia, Spain.

[#]Corresponding author: Ana Hurtado (ahurtado@neiker.eus)

Keywords: Extended-spectrum β-lactamase (ESBL) producing *E. coli*, AmpC β-lactamase producing *E. coli*, whole-genome sequencing (WGS), antimicrobial resistance (AMR), livestock, *bla*$_{CTX-M-65}$

Running title: IncHI2-mediated spread of *bla*$_{CTX-M-65}$ in livestock *E. coli*

Supplementary Note S1.

Surveillance of multidrug-resistant ESBL/AmpC-producing *Escherichia coli* from livestock at slaughter in northern Spain (Basque Country) in 2021-2023

Sample collection, isolation and antimicrobial susceptibility testing

Rectal feces (cattle, sheep and pigs) or caeca (free-range chicken) were collected from 445 animals and processed in pools of 5 animals (same animal species, slaughterhouse, and sampling date). Thus, a total of 89 pools were processed (51 cattle, 8 sheep, 15 chickens and 15 pigs). Samples (25 g of feces or 5 g of cecal content) were diluted 1:10 in buffered peptone water (BPW, bioMérieux) and incubated at 37ºC for 20±2 hours. For the isolation of ESBL/AmpC-producing *E. coli*, two loops (20 µl) of BPW were subcultured on selective MacConkey agar with cefotaxime (1 mg/L) and incubated at 37°C for 20±2 h. For the detection of carbapenemase (CP)-producing *E. coli*, two loops (20 µl) of BPW were subcultured on MacConkey agar (bioMérieux) without antimicrobials and incubated at 37ºC for 20±2 h. A loopful of grown colonies was then harvested for DNA extraction (Instagene kit, Bio-Rad) and subjected to real-time PCR amplification screening targeting the CP-encoding genes bla_{NDM}, bla_{VIM}, bla_{KPC}, and $bla_{\mathrm{OXA\text{-}48}}$ (Ellington et al., 2016). Samples positive to any of these genes were subcultured on ChromID® Carba Smart selective agar (bioMérieux) for isolation. Isolates compatible with a β-lactamase and/or CP-producing *E. coli* profile were stored at -80°C for further analysis.

Phenotypic resistance profiles of ESBL/AmpC-producing *E. coli* isolates were determined by minimum inhibitory concentration (MIC) determination using the Sensititre® microdilution plate system (Thermo Fisher Scientific). Presumptive ESBL-, AmpC- or CP-producing *E. coli* isolates obtained from slaughtered animals were initially tested with the EUVSEC3 panel (Thermo Fisher Scientific) that contains 15 antimicrobial agents (nine classes). All isolates with resistance to cefotaxime, ceftazidime or meropenem in the EUVSEC3 panel were subsequently tested with the EUVSEC2 panel (Thermo Fisher Scientific) for the phenotypic characterization of presumptive ESBL, AmpC, and CP producers. For interpretation of MIC (microbiological susceptibility or resistance to a given antibiotic), epidemiological cut-off values (ECOFF) were used as indicated by EUCAST (European Committee of Antimicrobial Susceptibility testing - EUCAST, https://www.eucast.org/).

ESBL/AmpC-producing *E. coli* isolation rates and antimicrobial susceptibility

No CP-producing *E. coli* were detected by PCR screening of the loopful of bacterial growth recovered from non-selective MacConkey agar (without antibiotics) confirming that they do not seem to be circulating in ruminant herds in the Basque Country. Similarly, CP-producing *E. coli* were not detected in a cross-sectional survey conducted in the Basque Country in 2014–2016 in 300 ruminant herds (Tello

et al., 2020), and a single CP-producing *E. coli* (Tello, Oporto, *et al.*, 2022) was detected after monitoring five dairy cattle herds for over a 2-year period (Tello, Ocejo, *et al.*, 2022), which suggests that their presence is sporadic. This is in agreement with data reported in Europe (Köck *et al.*, 2018; EFSA/ECDC, 2025; Hernández *et al.*, 2025).

In contrast, growth on cefotaxime-supplemented MacConkey agar was observed in 42/89 pools (47.2%), confirming that ESBL/AmpC-producing *E. coli* are widespread in livestock in northern Spain (Tello *et al.*, 2020). Using pairwise comparisons of proportions with two-sided Fisher's exact tests, the proportion of positive pools was significantly higher in pigs (13/15) than in cattle (22/51; $p = 0.003$), chickens (5/15; $p = 0.007$), and sheep (2/8; $p = 0.006$). Fifty-one isolates identified as ESBL/AmpC-producing *E. coli* (a maximum of two isolates from each positive pool) were subjected to MIC-based AST resulting in 31 different phenotypic resistance profiles (based on resistance to 20 antimicrobials) (Fig. 1). Eighteen of these profiles were unique; the remaining were shared by 2-5 isolates recovered from 1-3 animal species.

Figure 1. Phenotypic antimicrobial resistance profiles of ESBL/AmpC-producing *E. coli* isolates from livestock. UpSet plot showing 31 unique phenotypic resistance profiles among isolates from healthy cattle (n=22), pigs (n=15), sheep (n=4), and chickens (n=10). Bar height indicates the number of isolates exhibiting each profile for each host, which is color-coded as the legend. The matrix below represents combinations of resistance to individual antibiotics. On the left, a table summarizes the percentage of resistance per antibiotic for each animal host species.

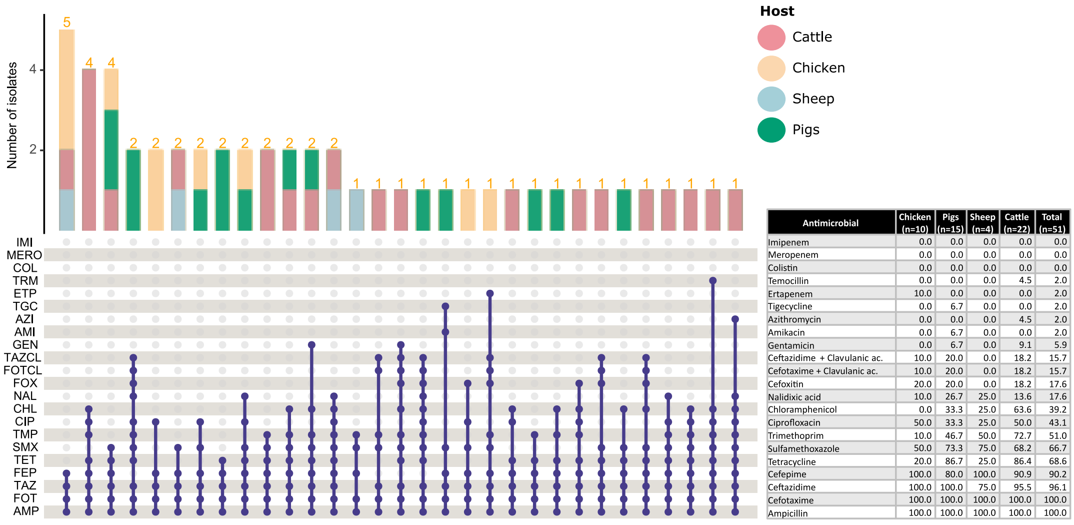

Antimicrobial	Chicken (n=10)	Pigs (n=15)	Sheep (n=4)	Cattle (n=22)	Total (n=51)
Imipenem	0.0	0.0	0.0	0.0	0.0
Meropenem	0.0	0.0	0.0	0.0	0.0
Colistin	0.0	0.0	0.0	0.0	0.0
Temocillin	0.0	0.0	0.0	4.5	2.0
Ertapenem	10.0	0.0	0.0	0.0	2.0
Tigecycline	0.0	6.7	0.0	0.0	2.0
Azithromycin	0.0	0.0	0.0	4.5	2.0
Amikacin	0.0	6.7	0.0	0.0	2.0
Gentamicin	0.0	6.7	0.0	9.1	5.9
Ceftazidime + Clavulanic ac.	10.0	20.0	0.0	18.2	15.7
Cefotaxime + Clavulanic ac.	10.0	20.0	0.0	18.2	15.7
Cefoxitin	20.0	20.0	0.0	18.2	17.6
Nalidixic acid	10.0	26.7	25.0	13.6	17.6
Chloramphenicol	0.0	33.3	25.0	63.6	39.2
Ciprofloxacin	50.0	33.3	25.0	50.0	43.1
Trimethoprim	10.0	46.7	50.0	72.7	51.0
Sulfamethoxazole	50.0	73.3	75.0	68.2	66.7
Tetracycline	20.0	86.7	25.0	86.4	68.6
Cefepime	100.0	80.0	100.0	90.9	90.2
Ceftazidime	100.0	100.0	75.0	95.5	96.1
Cefotaxime	100.0	100.0	100.0	100.0	100.0
Ampicillin	100.0	100.0	100.0	100.0	100.0

In addition to resistance to β-lactams, the proportion of isolates with resistance associated with folate pathway antagonists (trimethoprim and sulfamethoxazole) and tetracycline were very high in cattle, sheep and pigs, and high and moderate, respectively, in chickens. Resistance to ciprofloxacin was more frequent than to

nalidixic acid, particularly among cattle and chicken isolates. The proportion of chloramphenicol-resistant isolates was higher in cattle compared to sheep and pigs but was not detected in chicken isolates. Resistance to gentamicin, amikacin, azithromycin, tigecycline, temocillin and ertapenem was sporadic (one or two isolates), and all isolates were susceptible to colistin, imipenem and meropenem. Overall, 72.5% of isolates (37/51) were classified as multi-drug resistant (MDR), i.e., resistant to β-lactams and at least two other antimicrobial classes. The highest proportions of MDR strains were found in pigs (86.7%) and cattle (86.4%), with chicken and sheep isolates having lower MDR rates (40.0% and 25.0%, respectively) (Fig. 2).

Figure 2. Distribution of the phenotypic antimicrobial class resistance based on broth microdilution in ESBL/AmpC-producing E. coli from healthy livestock. Stacked bar chart shows the proportion of isolates from each host according to the number of different antimicrobial classes to which they were resistant (ranging from 1 to 6).

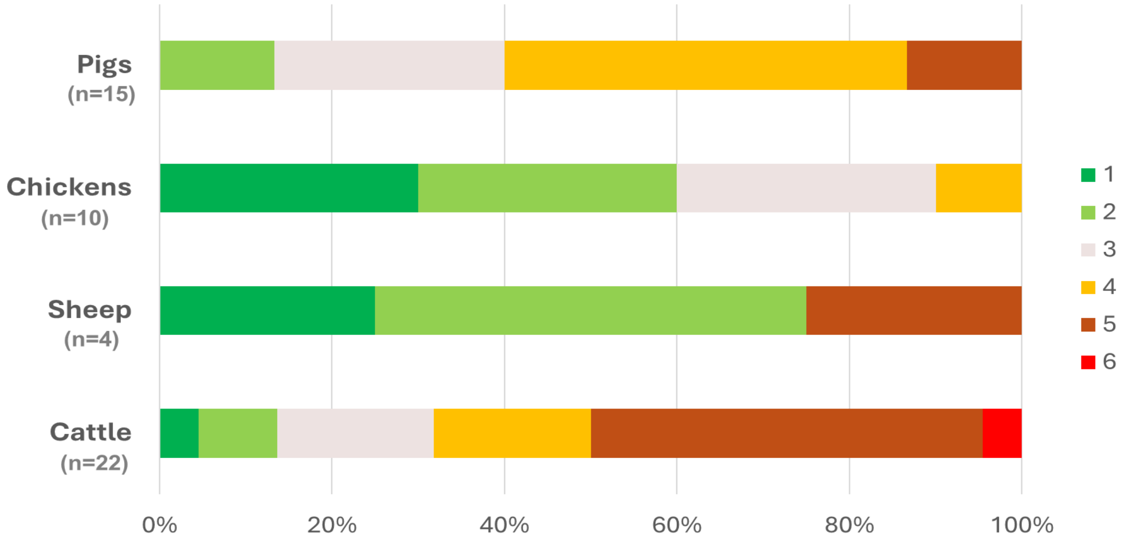

The lower proportion of MDR isolates in chickens compared to other studies (von Tippelskirch *et al.*, 2018; Damianos *et al.*, 2025) could be due to the fact that the chickens sampled in this study were reared in a free-range farming system with very limited use of antimicrobials. ESBL/AmpC-producing *E. coli* are commonly co-resistant to other classes of antimicrobials (Seiffert *et al.*, 2013; Köck *et al.*, 2018) due to the presence of other ARGs, often in the same plasmid carrying the ESBL-encoding genes. Co-selection associated with antimicrobial use and dissemination via MGEs contributes to maintaining ESBL-producing populations.

References

Damianos, A., Tsitsos, A., Economou, V., Gioula, G. and Haidich, A.B. (2025) "Systematic review and meta-analysis of the occurrence of ESBL-producing *Escherichia coli* and *Salmonella* spp. in foods of animal origin in Europe," *Food Control*, 171, p. 111127. Available at: https://doi.org/10.1016/J.FOODCONT.2024.111127.

EFSA/ECDC (2025) "The European Union summary report on antimicrobial resistance in zoonotic and indicator bacteria from humans, animals and food in 2022–2023," *EFSA Journal*, 23(3), p. e9237. Available at: https://doi.org/10.2903/J.EFSA.2025.9237.

Hernández, M., Falcó-Prieto, Á., Ugarte-Ruiz, M., Miguela-Villoldo, P., Ocampo-Sosa, A., Abad, D., Pérez-Sancho, M., Álvarez, J., Cadamuro, R.D., Elois, M.A., Fongaro, G., Quesada, A., González-Zorn, B., Domínguez, L., Eiros, J.M. and Rodríguez-Lázaro, D. (2025) "Genome analysis of 6222 bacterial isolates from livestock and food environments in Spain to decipher the antibiotic resistome," *Antibiotics*, 14(3), p. 281. Available at: https://doi.org/10.3390/ANTIBIOTICS14030281.

Köck, R., Daniels-Haardt, I., Becker, K., Mellmann, A., Friedrich, A.W., Mevius, D., Schwarz, S. and Jurke, A. (2018) "Carbapenem-resistant Enterobacteriaceae in wildlife, food-producing, and companion animals: a systematic review," *Clinical Microbiology and Infection*, 24(12), pp. 1241–1250. Available at: https://doi.org/10.1016/J.CMI.2018.04.004.

Seiffert, S.N., Hilty, M., Perreten, V. and Endimiani, A. (2013) "Extended-spectrum cephalosporin-resistant gram-negative organisms in livestock: An emerging problem for human health?," *Drug Resistance Updates*, 16(1–2), pp. 22–45. Available at: https://doi.org/10.1016/j.drup.2012.12.001.

Tello, M., Ocejo, M., Oporto, B. and Hurtado, A. (2020) "Prevalence of cefotaxime-resistant *Escherichia coli* isolates from healthy cattle and sheep in Northern Spain: Phenotypic and genome-based characterization of antimicrobial susceptibility," *Applied and Environmental Microbiology*, 86(15), pp. e00742-20. Available at: https://doi.org/10.1128/AEM.00742-20.

Tello, M., Ocejo, M., Oporto, B., Lavín, J.L. and Hurtado, A. (2022) "Within-farm dynamics of ESBL-producing *Escherichia coli* in dairy cattle: Resistance profiles and molecular characterization by long-read whole-genome sequencing," *Frontiers in Microbiology*, 13, p. 936843. Available at: https://doi.org/10.3389/FMICB.2022.936843.

Tello, M., Oporto, B., Lavín, J.L., Ocejo, M. and Hurtado, A. (2022) "Characterization of a carbapenem-resistant *Escherichia coli* from dairy cattle harbouring blaNDM-1in an IncC plasmid," *Journal of Antimicrobial Chemotherapy*, 77(3), pp. 843–845. Available at: https://doi.org/10.1093/jac/dkab455.

von Tippelskirch, P., Gölz, G., Projahn, M., Daehre, K., Friese, A., Roesler, U., Alter, T. and Orquera, S. (2018) "Prevalence and quantitative analysis of ESBL and AmpC beta-lactamase producing Enterobacteriaceae in broiler chicken during slaughter in Germany," *International Journal of Food Microbiology*, 281, pp. 82–89. Available at: https://doi.org/10.1016/J.IJFOODMICRO.2018.05.022.

Supplementary Note S2.

Comparative analysis of ESBL/AmpC-producing *E. coli* isolates from different animal sources: Genotypic resistance in healthy animals was more widespread in cattle

Differences among *E. coli* isolates from different animal hosts in terms of the total number of GDRs and the number of antimicrobial classes for which these GDRs encoded resistance were evaluated using the Kruskal–Wallis test, followed by Dunn's post hoc pairwise comparisons with FDR correction. Cattle isolates carried the highest total number of GDRs, whereas isolates from chicken carried the lowest (Fig. 3A); a similar pattern was observed for the number of antimicrobial classes for which they encoded resistance (Fig. 3B). This distribution of GDR correlates with the phenotypic resistance profiles observed. The lower resistance burden in chicken isolates was mainly explained by the less frequent detection of genetic determinants associated with resistance to aminoglycosides, folate pathway antagonists, and tetracycline, as well as the absence of macrolides, lincosamides, phenicols, or rifamycins (Fig. 4). ARG-carrying plasmids were detected in most healthy-animal isolates, but the number of ARGs per ARG-carrying plasmid was significantly higher in cattle (median = 5 ARGs/plasmid) than in chickens and pigs (median = 2 for both; Kruskal–Wallis test followed by Dunn's post hoc pairwise comparisons with FDR correction, $p = 0.020$).

Figure 3. Distribution and comparison of genetic determinants of resistance (GDRs) and associated antimicrobial class resistance in ESBL/AmpC-producing *E. coli* from different livestock species. Violin plots showing the distribution of the total of GDRs per isolate **(A)** and the number of antimicrobial classes associated with the GDRs detected in each isolate **(B)**. Box plots are overlaid to show distribution and spread, with red dots indicating median values. Results for non-parametrical Kruskal–Wallis or Mann–Whitney test is provided. If applicable, Dunn's post hoc test results (*p*-values) are shown for significant pairwise comparisons.

A)

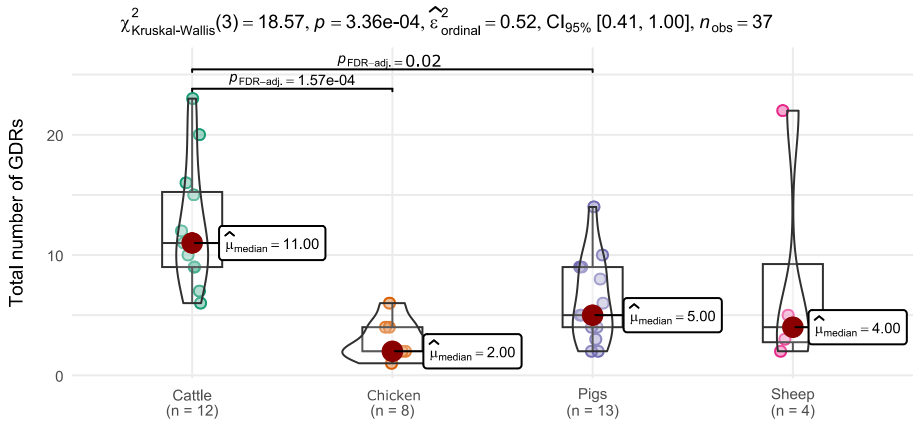

B)

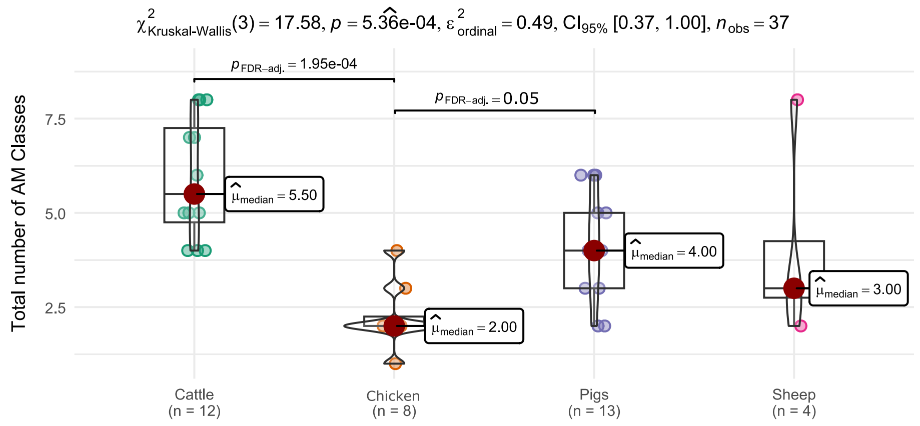

Figure 4. Genotypic resistance to antimicrobial classes among ESBL/AmpC-producing *E. coli* isolates from healthy livestock (cattle, chickens, pigs and sheep). The matrix of dots marks the predicted resistance for an antimicrobial class based on presence of associated GDR. Each vertical bar represents a unique combination of genotypic resistance profile, with the height indicating the number of isolates carrying that particular combination and the color indicates the host, which is coded as the legend. The table on the right summarizes the proportion (%) of isolates from each host with genotypic resistance genes for each antimicrobial class.

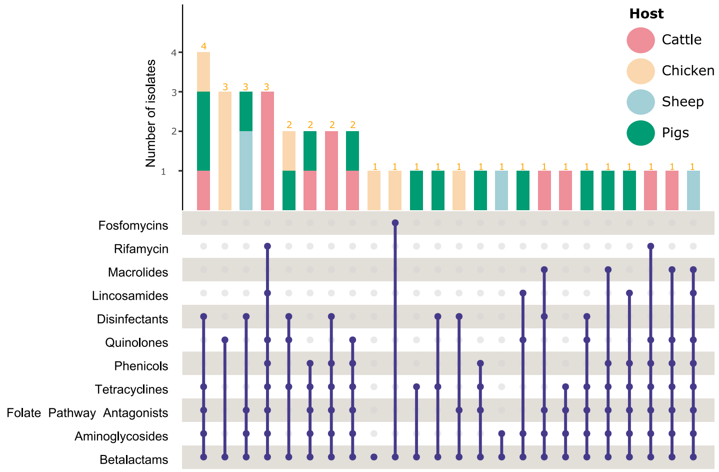

Chicken	Pigs	Sheep	Cattle	All
12.5	0.0	0.0	0.0	2.7
0.0	0.0	0.0	33.3	10.8
0.0	15.4	25.0	25.0	16.2
0.0	7.7	25.0	16.7	10.8
37.5	46.2	50.0	33.3	40.5
0.0	38.5	25.0	75.0	40.5
50.0	30.8	25.0	50.0	40.5
25.0	84.6	25.0	91.7	67.6
25.0	69.2	75.0	100.0	70.3
12.5	69.2	100.0	100.0	70.3
100.0	100.0	100.0	100.0	100.0

Supplementary Note S3.

Comparative analysis of ESBL/AmpC-producing *E. coli* isolates from healthy and diseased cattle: Genotypic resistance was more widespread in diseased cattle than in healthy cattle

Clinical isolates from diseased cattle carried a significantly higher total number of individual GDRs than isolates from healthy cattle (median = 17 vs. 11; Mann–Whitney U test, $p = 0.030$) (Fig. 5A). When considering the number of antimicrobial classes for which those individual GDRs encoded resistance, the number of antimicrobial classes represented per isolate was also slight but non-significantly different between isolates from diseased and healthy cattle (median = 7 vs. 5.5 classes, Mann–Whitney U test, $p = 0.330$) (Fig. 5B). Only quinolone-associated GDRs were significantly more frequent in isolates from diseased than healthy cattle (92.3% vs. 50%; Fisher's exact test with FDR correction, OR = 11.1, 95% CI [1.52 – 100], $p = 0.007$), whereas no differences were found for GDRs associated with resistance to aminoglycosides, tetracyclines, and folate antagonists (Fig. 6).

Figure 5. Distribution and comparison of genetic determinants of resistance (GDRs) and associated antimicrobial class resistance in ESBL/AmpC-producing *E. coli* from healthy and diseased cattle. Violin plots showing the distribution of the total number of GDRs per isolate (A) and the number of antimicrobial classes associated with the GDRs detected in each isolate (B). Box plots are overlaid to show distribution and spread, with red dots indicating median values. Results for non-parametrical Kruskal–Wallis or Mann–Whitney test is provided. If applicable, Dunn's post hoc test results (*p*-values) are shown for significant pairwise comparisons.

A)

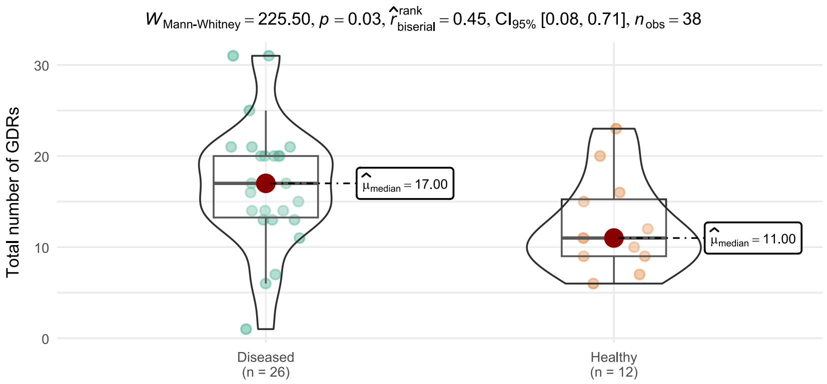

B)

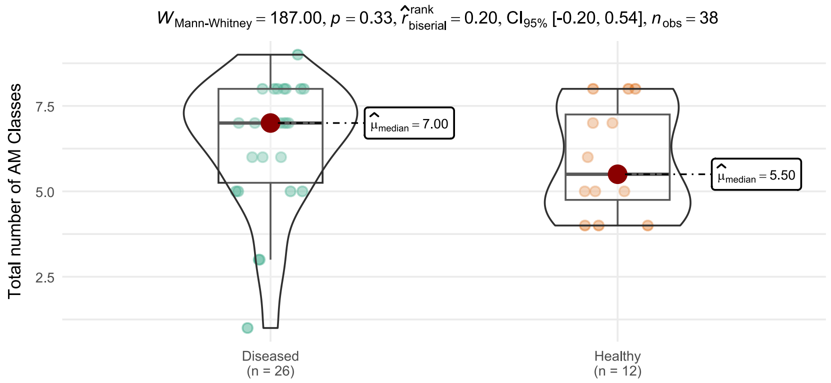

Figure 6. Genotypic resistance to antimicrobial classes among ESBL/AmpC-producing *E. coli* isolates from healthy and diseased cattle. The matrix of dots marks the predicted resistance for an antimicrobial class based on presence of associated GDR. Each vertical bar represents a unique combination of genotypic resistance profile, with the height indicating the number of isolates carrying that particular combination and the color indicates the host, which is coded as the legend. The table on the right summarizes the proportion (%) of isolates from each host with genotypic resistance genes for each antimicrobial class.

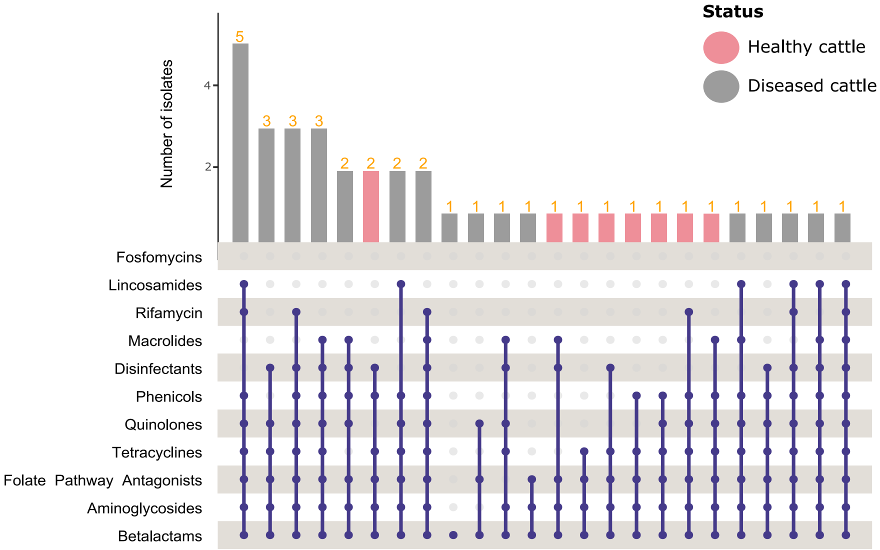

Healthy Cattle	Diseased Cattle
0.0	0.0
33.3	34.6
25.0	30.8
16.7	42.3
33.3	69.2
75.0	73.1
50.0	92.3
91.7	80.8
100.0	92.3
100.0	92.3
100.0	100.0
